# The Prevalence of Food Addiction as Assessed by the Yale Food Addiction Scale: A Systematic Review

**DOI:** 10.3390/nu6104552

**Published:** 2014-10-21

**Authors:** Kirrilly M. Pursey, Peter Stanwell, Ashley N. Gearhardt, Clare E. Collins, Tracy L. Burrows

**Affiliations:** 1 School of Health Sciences, Priority Research Centre for Physical Activity and Nutrition, University of Newcastle, Callaghan, NSW 2308, Australia; E-Mails: kirrilly.pursey@newcastle.edu.au (K.M.P.); clare.collins@newcastle.edu.au (C.E.C.); 2 School of Health Sciences, Priority Research Centre for Translational Neuroscience and Mental Health, University of Newcastle, Callaghan, NSW 2308, Australia; E-Mail: peter.stanwell@newcastle.edu.au; 3 Department of Psychology, University of Michigan, Ann Arbor, MI 48109, USA; E-Mail: agearhar@umich.edu

**Keywords:** food addiction, Yale Food Addiction Scale, YFAS, obesity, eating disorders, substance dependence, addiction

## Abstract

Obesity is a global issue and it has been suggested that an addiction to certain foods could be a factor contributing to overeating and subsequent obesity. Only one tool, the Yale Food Addiction Scale (YFAS) has been developed to specifically assess food addiction. This review aimed to determine the prevalence of food addiction diagnosis and symptom scores, as assessed by the YFAS. Published studies to July 2014 were included if they reported the YFAS diagnosis or symptom score and were published in the English language. Twenty-five studies were identified including a total of 196,211 predominantly female, overweight/obese participants (60%). Using meta-analysis, the weighted mean prevalence of YFAS food addiction diagnosis was 19.9%. Food addiction (FA) diagnosis was found to be higher in adults aged >35 years, females, and overweight/obese participants. Additionally, YFAS diagnosis and symptom score was higher in clinical samples compared to non-clinical counterparts. YFAS outcomes were related to a range of other eating behavior measures and anthropometrics. Further research is required to explore YFAS outcomes across a broader spectrum of ages, other types of eating disorders and in conjunction with weight loss interventions to confirm the efficacy of the tool to assess for the presence of FA.

## 1. Introduction

Obesity has been described as a global epidemic with 36.9% males and 38.0% females worldwide classified as overweight or obese [1]. This is significant given the increased risk of chronic conditions associated with obesity such as cardiovascular disease and type 2 diabetes [2], as well as psychological implications including decreased quality of life and weight related social stigma [3]. It has been suggested that an addiction to certain types of food, particularly highly processed, hyper-palatable foods, could be a factor contributing to overeating and obesity in parallel with dramatic changes in the food environment [4]. Negative perceptions similar to those related to obesity are now associated with food addiction (FA) [5], but interestingly, obesity-related stigma is reduced when it is framed in the context of FA [6].

The term “food addiction” has been used in combination with specific eating behaviors to describe an abnormal pattern of excessive consumption [7,8,9]. While behavioral addictions such as gambling have been recently recognized by the Diagnostic and Statistical Manual of Mental Disorders (DSM) [10], there is no consensus that FA is a clinical disorder nor is there a universally accepted definition for FA. A widely used definition for FA has emerged by mapping the DSM-IV diagnostic criteria for substance dependence to eating behaviors [9]. These include: tolerance, withdrawal symptoms, larger amounts consumed than intended, persistent desire or unsuccessful attempts to cut down, much time spent using or recovering from substance, continual use despite knowledge of consequences, activities given up due to use of substance [10]. While neuroimaging techniques have become a popular method to explore FA, only one neuroimaging study has investigated the FA phenotype as defined by the DSM substance dependence criteria [11]. This study identified similarities in neural responses between addictive-like eating and traditional addiction. While there have been many more neuroimaging studies of obesity as a proxy for FA [12,13,14,15,16], findings have been inconsistent [17]. This may be because obesity is a heterogeneous condition and it is unclear as to the proportion of obese participants included in these studies who are truly addicted to certain foods. There is, however, preliminary evidence that dopaminergic brain circuits commonly associated with substance dependence are also implicated in abnormal eating behaviors such as overeating in obesity [18,19]. It is therefore possible that a physiological addiction to food underpinned by neural mechanisms could help to explain some of the inefficacy of current weight programs focusing on diet and exercise [20].

Despite an increase in the number of publications regarding FA [17], with a PubMed search of “food addiction” identifying 809 publications in the past five years alone, little attention has been paid to the clinical assessment of FA. Terms synonymous with FA, such as “food addict” “chocoholic” and “carb craver”, have been used in lay literature for decades. However, the assessment of FA has largely relied on self-identification, used elevated BMI as a proxy for FA or administered non-validated tools with no evidence to support the use of specific assessment measures [4]. This has led to variation in reports of FA prevalence, with a lack of characterization of the FA construct within surveys and potential misclassification of individuals who could be considered as food addicted. A variety of self-reported questionnaires has been used to assess addictive food and eating tendencies. Existing tools such as the Food Craving Questionnaire [21,22], Dutch Eating Behavior Questionnaire [23], Three Factor Eating Questionnaire [24], and Power of Food Scale [25], have investigated possible characteristics related to addictive eating such as restraint, disinhibition, impulsivity, and craving. However, these addictive-like behaviors have usually been studied in isolation.

A tool specifically designed to assess FA, the Yale Food Addiction Scale (YFAS) [26], was developed in 2009 by modelling all of the DSM-IV for substance dependence to be applicable to eating behavior. The development of the YFAS has allowed for the exploration of potential FA across populations using a standardized tool. Previous research has demonstrated that the YFAS has sound psychometric properties including adequate internal consistency (original validation study α = 0.86), as well as convergent, discriminant, and incremental validity [26,27]. The YFAS uses two scoring options including a FA symptom score and diagnosis. Participants are allocated a symptom score from zero to seven corresponding with the number of DSM-IV diagnostic criteria endorsed. Additionally, a “diagnosis” of FA is assigned to participants who endorse three or more symptoms plus satisfying the clinical impairment criteria, in line with the DSM-IV diagnosis of traditional substance dependence.

To the authors’ knowledge, only one study to date has provided an overview of how the YFAS has been used to measure FA [28]. No reviews to date have systematically examined studies that have used the YFAS. Given that FA is a rapidly growing area of research and the YFAS is the only currently available tool to assess FA, it is timely to review how the tool has been used and applied in research and practice. This study aimed to systematically review studies which have used the YFAS to assess FA and its related symptoms and to subsequently conduct a meta-analysis of study outcomes. The primary outcome of the review was to determine the prevalence of FA diagnosis and symptom sub-scales across a variety of study populations. Other outcomes of the review were to determine the prevalence of FA by age group, weight status, and gender to identify whether specific groups may be more predisposed to FA, and to determine if there are any relationships between the YFAS and other eating related variables.

## 2. Methods

A systematic literature review was undertaken to identify published studies that used the YFAS to assess FA diagnosis or symptom score from the year of tool development, 2009, to July 2014.

Electronic databases were searched to identify relevant publications. These included: MEDLINE, The Cochrane Library, EMBASE (Excerpta Medica Database), CINAHL (Cumulative Index to Nursing and Allied Health), Informit Health Collection, Proquest, Web of Science, Scopus and PsycINFO. Keywords were informed by preliminary literature searches and were searched including: Yale Food Addiction Scale, YFAS, questionnaire; food addiction, behavioral addiction, eating behavior, obesity, food, eat, feeding behavior, food preferences, food habits, body mass index, overeat, hyperphagia, substance related disorders, binge eating, hedonic eating. Both the English and American spellings of behavior/behaviour were searched. Database searches were supplemented by cited reference checks and systematic checking of reference lists of identified articles for additional relevant publications. The search strategy was registered with PROSPERO [29].

To determine eligibility for inclusion in the review, titles and abstracts of identified studies were assessed by two independent reviewers using a predetermined inclusion criterion. Studies were included if they used the YFAS or a modified form of the YFAS to assess FA, reported either the YFAS diagnosis or symptom score, reported the population demographics in detail and were published in the English language. The articles for all studies meeting the inclusion criteria were retrieved. If a study’s eligibility for inclusion was unclear, the article was retrieved for further clarification.

Quality of retrieved studies was assessed by two independent reviewers using a standardized 9-item tool [30]. The quality criteria included items such as the method of sample selection, ways of dealing with confounding factors, reliability of outcome measures, and statistical analysis. Each item was classified as present “Yes”, absent “No” or “Unclear” for each included study and then each response recoded as +1, 0 and −1 respectively. Items were classified as “non-applicable” if the item was not relevant to the study design and was scored as 0. High quality studies were deemed to have a score of eight or above out of a maximum score of nine. No studies were excluded based on quality ratings. Data were extracted using standardized tables developed for the review. In cases of uncertainty of a study’s inclusion, a third independent reviewer was consulted until consensus was reached.

Studies were tabulated in chronological order. Results are reported based on scoring options used including: diagnosis of FA, YFAS symptom score and studies that reported high and low FA scores. Studies were grouped by BMI, age and gender for comparison in the systematic review and meta-analysis. As only two studies reported the prevalence of FA diagnosis of a sample with a mean BMI in the overweight category, studies of overweight or obese participants were grouped in a single category for meta-analysis. Participants were classified as healthy weight if mean BMI <25 kg/m^2^, or classified as overweight/obese if mean BMI ≥25 kg/m^2^. Participants were classified as children and adolescents (<18 years), young adults (18–35 years), and older adults (>35 years) to control for possible age related differences related to life stage (e.g., marital status and household structure) as well as dietary patterns and nutrient intakes [31]. Where BMI or age ranged across numerous categories, mean BMI or age was used to classify participants into a single category. If studies reported the prevalence of FA diagnosis for a number of weight status categories separately, YFAS outcomes for the specific weight category were entered into the respective analysis. Although one study reported YFAS outcomes for adults aged >65 years separately, data for this study was entered as a single data point in the meta-analysis to remain consistent across studies. Participants were also grouped by clinical status for meta-analysis. For the meta-analysis of FA diagnosis, participants were grouped as having a current clinically diagnosed eating disorder (e.g., binge eating disorder (BED), bulimia nervosa) as non-disordered eating if a no diagnosis of an eating disorder was present. Additionally, for meta-analysis of symptom scores, participants were classified as a clinical population if they were recruited from a clinical setting or had a current diagnosis of an eating disorder, or as a non-clinical sample if they did not meet these criteria.

Results were pooled using meta-analysis if the study reported the proportion of individuals with a diagnosis or mean symptom score as well as the number of participants. Due to the limited number of studies and lack of standardized definition for studies reporting high and low FA groups, only diagnosis and symptom score were included in the meta-analysis. Heterogeneity was tested during meta-analysis and if significant heterogeneity was present, the random effects model was used for statistical analysis. Sub-analysis by sex (male or female), weight status (healthy weight, overweight or obese), age group (young adults 18–35 years or older adults >35 years) and clinical status (clinical *vs.* non-clinical population) was also undertaken if there were enough studies to conduct separate meta-analyses. As only one study reported FA prevalence for children, this study was not included in the meta-analysis. Meta-analyses were conducted using Comprehensive Meta-Analysis Professional version 2 (Biostat, Inc., Englewood, NJ, USA). If details were not reported, authors were contacted in an attempt to gain the information required.

The authors acknowledge that there is no universally accepted definition for FA, however, terms such as “food addicted” and “diagnosis” are used for brevity in subsequent sections of the paper and refer to the criteria used to diagnose FA as stipulated by the YFAS.

## 3. Results

**Figure 1:**
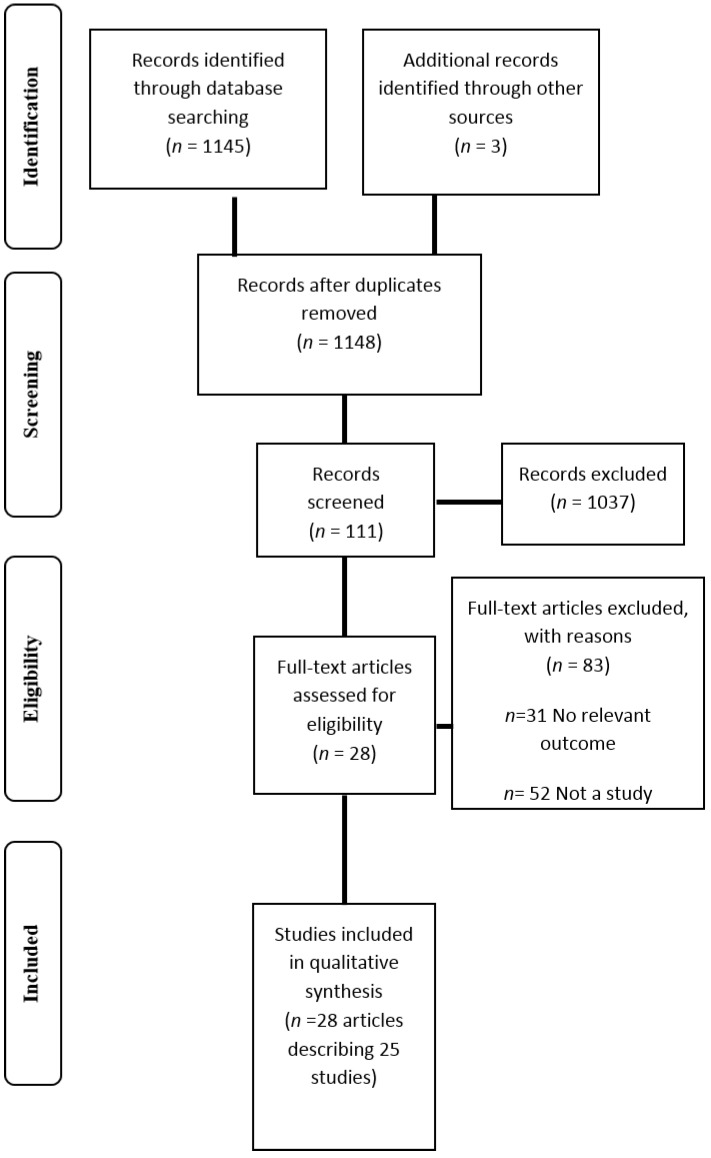
Flow diagram of studies included in the review.

A total of 1148 articles were initially identified using the search strategy. Following the removal of duplicate references and assessment of articles using the predefined inclusion criterion, 28 relevant articles describing 25 studies were identified (Figure 1) [11,26,27,32,33,34,35,36,37,38,39,40,41,42,43,44,45,46,47,48,49,50,51,52,53,54,55,56,57]. Primary reasons for exclusion included the article being narrative in nature and the study including no outcomes relevant to the review. The majority of studies were published from 2013 onwards (*n* = 18) [32,33,34,35,36,37,38,39,40,41,43,44,45,46,47,48,49,50,51] and in the United States (*n* = 15) [11,26,27,33,35,36,38,39,43,44,45,46,48,49,50]. As shown in Table 1, all studies were cross sectional in design excluding three [34,44,52], and only one study assessed outcomes of the YFAS at more than one time point [34]. Eight studies included individuals seeking or participating in weight loss treatment [11,27,37,38,39,45,47,49], while three studies included bariatric surgery candidates [44,46,56]. Four studied individuals with a diagnosed eating disorder including BED and bulimia nervosa [27,32,36,49]. Four studies reported a follow up assessment period following the completion of the YFAS (seven weeks to nine months) [38,39,44,45,52]. Only one study of these studies assessed and reported outcomes of the YFAS at baseline and follow up after nine months [34].

Critical appraisal of the quality of included studies is described in Table 2. Of the nine quality items, only one study was classified as high quality (a score above eight) using the pre-defined quality scoring [35]. Eight studies had a quality score below four. Quality criteria including the control of confounders and handling of withdrawals was poorly described across reviewed studies. Only five of the 25 studies described characteristics of participants who were not included in the final study sample and only fifteen studies described controlling for potential confounding variable in detail. The criteria assessing adequacy of follow-up period was not-applicable to all studies excluding three, which may be attributable to the overwhelming number of cross-sectional studies included in the review.

**Table 1:** Characteristics of included studies using the Yale Food Addiction Scale (YFAS) to assess food addiction.

Author, year, country	Type of study	Number of participants	Population studied	Participant characteristics	Study information	Outcome measures	YFAS details (e.g., Type and method of administration)	Symptom score or diagnosis used?	Follow up? Duration?	Retention rate
**Brunault, 2014, France** [32]	Cross sectional	*n* = 553	Non clinical sample	Age: 28.9 ± 12.0 years (95% CI 27.9–29.9 years). BMI: 22.5 ± 4.5 kg/m^2^ (95% CI 22.2–22.9 kg/m^2^).	Assessment of the psychometric properties of the French version of the YFAS.	Socio demographic characteristics; BMI; BES; the Bulimic Investigatory Test- Edinburgh	YFAS French version. Online questionnaire	D + SS	N/A	N/A
**Burgess, 2014, USA** [33]	Cross sectional	*n* = 150	College undergraduate students	70.7% female Age: 24.4 years (range 17–60) BMI: 26.3 (range 16.4–51.0 kg/m^2^). 2% underweight, 52.7% HW, 22% OW, 18% OB, 5.3% severely OB.	Validation of the Palatable Eating Motives Scale.	PEMS; BES; Sensitivity to punishment and reward; BMI; Demographics	YFAS. Paper based survey completed at face to face session.	SS	N/A	N/A
**Davis 2014, Canada** [34]	Double blind crossover design	*n* = 136	Healthy adults participating in a community based study of overeating	Age: 25–50 years 67.6% female Age food addicts 33.9 ± 5.9 years, non-food addicts 32.4 ± 6.6 years (NS). BMI: food addiction group 34.6 ± 7.0 kg/m^2^, non-food addiction group 33.8 ± 8.4 (NS). 26% food addicts and 20% control group smokers.	Part of a larger study. 3 assessment sessions Participants randomised to oral methylphenidate or placebo capsule. Methylphenidate suppresses appetite and reduces binge episodes. Participants could eat as much of snack food as desired 3 h post capsule	General FCQ- State; Appetite ratings, Snack food consumption; Demographics; Favorite snack foods; mood ratings.	Questionnaires distributed at initial session and completed at home.	D	3 sessions, 1 week interval.	100%
**Flint, 2014, USA** [35]	Cross sectional	*n* = 134,175 YFAS and modified YFAS on subsample *n* = 2061.	Female nurses in the USA involved in the NHS and NHS II	100% female Age range: NHS 62–88 years, NHS II 45–64 years. BMI: NHS 43.7% HW, 34.2% OW, 22.2% OB. NHS II 40.2% HW, 30.5% OW, 29.2% OB. 86.6% NHS and 81.0% NHS II provided sufficient data	Biennial questionnaires. NHS established in 1976 with 121,700 nurses. Food addiction data collected 2008–2010. NHS II established in 1989 with 116,609 nurses. Food addiction data collected 2009–2011.	Demographics; Self-reported anthropometrics; Physical activity; Medical assessment	Modified YFAS NHS completed in written hardcopy, NHS II completed online.	D	N/A	N/A
**Gearhardt, 2014, USA** [36]	Cross sectional	*n* = 815	Non clinical sample of community volunteers	88.1% female Age: 33 years (range 18–73) BMI: 28.70 ± 8.77 kg/m^2^ (range 14.60–69.23) 79.1% Caucasian	Investigate the relationships between YFAS and BMI, BN and BED. Grouped based on eating behavior: (1) FA only; (2) BED/BN exclusively; (3) BED + FA; (4) BN + FA; (5) healthy controls no FA.	Demographics, self-reported anthropometrics, Eating Disorder Examination Questionnaire, Questionnaire for Eating and Weight Patterns Revised.	YFAS. No details re completion.	D + SS	N/A	N/A
**Imperatori, 2014, Italy** [37]	Cross sectional	*n* = 112	OW and OB patients seeking low energy diet therapy	71.4% female Age: 43.46 ± 12.91 years (range 18–73). BMI: 32.09 ± 6.76 kg/m^2^ (range 25.04–53.04). 49% used tobacco in previous 6 months. Majority employed and educated.	Aim: to test the association between FA, psychopathology and binge eating	BES; Symptom Checklist-90- Revised; Sociodemographic and clinical history.	Assessed at study baseline. YFAS translated in to Italian by authors. Cronbach’s alpha = 0.87	D + SS	N/A	N/A
**Lent, 2014, USA. Eichen, USA, 2013**. [38,39]	Cross sectional	*n* = 178	OW/OB adults seeking/enrolled in behavioral weight loss treatment	Whole sample: 51.2 ± 11.7 years, 36.1 ± 4.8 kg/m^2^, 92.1% OB, 74.7% female, 69.1% African American. Study 1: *n* = 100, Age 55.6 ± 10.6 years, BMI 35.8 ± 5.3 kg/m^2^, 59% African American. Study 2: *n* = 78, Age 45.6 ± 10.6 years, BMI 36.4 ± 4.2 kg/m^2^, 82.1% African American. Completers were older than non-completers (*p* = 0.001).	Study 1: 6 month behavioral intervention for individuals with T2DM-randomised to portion controlled diet or self-management program. Study 2: enhanced behavioral treatment for emotional eating including portion control, mindfulness and emotion regulation	BDI; BMI; Demographics; Anthropometrics	YFAS. Scale adapted from “past year” to “past month”. Completed at baseline.	D + SS	6 month weight loss program	Non completers 2.2% males, 17.3% females (*P* = 0.01).
**Meule, 2014, Germany** [40]	Cross sectional/case control	*n* = 109	Females with current BN recruited from outpatient clinics, remitted BN and control group	*n* = 26 current BN, *n* = 20 remitted BN, *n* = 63 control. Age: current BN 25.23 ± 5.82years, remitted BN 25.55 ± 3.72 years, control 23.57 ± 4.20 years (NS). BMI: current BN 20.92 ± 1.92 kg/m^2^, remitted BN 21.92 ± 1.50 kg/m^2^, control 21.84 ± 2.65 kg/m^2^ (NS).	Measurement of food addiction in BN and associations between BN symptomology and FA.	Eating disorder examination; DEBQ; Borderline Symptom List of BPD; Brief Symptom Inventory; Centre for Epidemiologic Studies Depression Scale-Short; Socio demographics; Anthropometrics	YFAS German version. No info re completion	D + SS	N/A	N/A
**Meule, 2014, 2012, Germany** [41,42]	Cross sectional	*n* = 50	Female students	*n* = 82 screened 100% female Age; 22.3 ± 3.0 years (range 19–32). BMI 21.5 ± 2.7 g/m^2^. 18 trying to control their weight.	Go no go trials where respond to X Y stimuli base on type of image shown: (high cal sweet or savory food images or neutral household objects). No food, caffeine, nicotine, or alcohol 3 h before task. FCQ completed immediately after task, other questionnaires completed same day or within 1–2 weeks.	Centre for Epidemiologic studies depression scale; BMI; Dieting status; Perceived self-regulatory success in dieting scale (PSRS); BIS-short; FCQ-S; Stop signal task (reaction times, errors).	YFAS. Completed on same day or within 1–2 weeks of task	SS	N/A	N/A
**Murphy 2014, USA** [43]	Cross sectional	*n* = 233	Students at the University of Georgia	77% female Age: 19.65 ± 2.15 years (range 18–32) BMI: 22.78 ± 4.0 kg/m^2^ (range 16.09–47.82 kg/m^2^). Underweight 5%, 73% HW, 16% OW, 5% OB. 84% white	Goal to investigate the relationships between food addiction, impulsive personality traits and weight status.	Impulsive behavior scale; demographics; Anthropometrics	YFAS. Pencil and paper format.	D + SS	N/A	N/A
**Pepino 2014, USA** [44]	Longitudinal (pre post test)	*n* = 44	OB patients selected to undergo gastric bypass surgery	RYGB *n* = 25, LAGB *n* = 11, SG *n* = 8. Age: non-food addicted: 42.6 ± 10.9 years, food addicted: 43.2 ± 11.1 years. *n* = 39 female BMI pre surgery: non-food addicted 48.2 ± 8.2 kg/m^2^, food addicted 47.5 ± 8.0 kg/m^2^ (NS). BMI post-surgery: non-food addicted 38.5 ± 6.9 kg/m^2^, food addicted 37.7 ± 6.6 kg/m^2^ (NS). Non-food addicted 77% white, food addicted 86% white.	FA assessment pre and 9 months post gastric bypass surgery. Dietary counselling after surgery: liquid diet first week followed by 2–4 week progression to regular food 1000–1200 kcal/day. Dietitian involved- weekly phone or face to face weight monitoring, education and advice.	DEBQ (emotional, external and restrained eating); Food Craving Inventory	Assessed at baseline and following surgical weight loss of ~20%. Questionnaires administered in a private room onsite at the Washington University.	D + SS	9 months	9 non completers: 8 non-food addict 1 food addict
**Burmeister, 2013, USA** [45]	Cross sectional	*n* = 57	OW/OB individuals seeking weight loss/part of weight loss intervention	68.4% female Age: 47.4 ± 13.7 years BMI: 38.2 kg/m^2^ ± 8.1. 84.2% Caucasian 70.2% married 91.2% college education.	18 week behavioral weight loss intervention. Individuals who met goals randomised to a lower intensity treatment at week 7. Results of the current study focus on outcomes up to 7 weeks prior to randomisation.	Centre for Epidemiological Studies Depression Scale; BES; DEBQ; Eating Self Efficacy Scale; Anti-Fat Attitudes questionnaire; Weight Bias; Objectified Body Consciousness Scale Body Shame; Multidimensional Body Self Relations Questionnaire Anthropometrics	YFAS. Completed at baseline	SS	7 week weight loss intervention	89.5%
**Clark, USA, 2013** [46]	Cross sectional	*n* = 67	Post bariatric surgery patients recruited from surgery support group	62.7% female Age: 42.27 years (range 25–73). 59.7% RYGB. 86.6% Caucasian	Validation of the YFAS in a post weight loss surgery population. Questionnaires completed before surgery	Demographics; BES; BIS/BAS reactivity; Eating attitudes test; Emotional eating scale; Michigan assessment screening test for alcohol and drugs; Alcohol, smoking and substance screening test	YFAS. Online survey. Completed pre surgery.	D + SS	N/A	N/A
**Davis, 2013, Canada** [47]	Cross sectional	*n* = 120	Recruited for overeating/OW study	68.3% female Age: food addicts 34.7 ± 5.9 years (range 45–44), non food addicts 32.5 ± 6.6 years (range 25–47) (NS) BMI: Food addicts: 35.5 ± 7.3 kg/m^2^ (range 22–49.4), Non food addicts 33.1 ± 8.9 kg/m^2^ (range 19–60) (NS). 24% met diagnostic criteria for BED.	Investigate relationships between YFAS and genotypes.	Demographics; Anthropometrics; Multilocus genetic profile; PFS; Binge eating questionnaire; DEBQ (emotional eating); Eating behaviors questionnaire (snacking on sweets); FCQ	YFAS. Paper based, taken home to complete.	D	N/A	N/A
**Gearhardt 2013, USA** [48]	Cross sectional	*n* = 75	Children and their parents recruited from a larger study on family eating habits	*n* = 117 recruited 42.7% female. Age: 8.32 ± 2.78 years (range 4–16 years) Parents 65.1% Caucasian.	Validation study of the YFAS for children.	BMI percentile (parental report); Child eating behavior questionnaire.	YFAS-C. Parents could complete scale.	D + SS	N/A	N/A
**Gearhardt, 2013, USA** [49]	Cross sectional	*n* = 96	OB BED patients respondents to a treatment study.	75.8% female. Age: 44.88 ± 12.82 years (range 19–65). BMI: 38.30 ± 5.73 kg/m^2^. 45.3% Caucasian, 32.6% African American, 12.6% Hispanic. 74.7% had college education.	Explore incremental validity of YFAS in predicting binge eating behavior beyond other measures.	Eating disorder examination; BDI-II; Difficulties in emotion regulation scale (DERS); Questionnaire on eating and weight problems revised (QWEP-R)	YFAS. No information re completion	D + SS	N/A	N/A
**Mason, 2013, USA** [50]	Cross sectional	*n* = 57,321	Female registered nurses involved in the NHS II	75% response rate 100% female NHS II age 25–42 years.	Biennial questionnaires 2001 survey included questions about experiences of sexual and physical abuse. 2009 survey included modified version of the YFAS.	Sexual and physical abuse experienced in childhood or adolescence (2001); Food addiction status (2009)	Modified YFAS.	D	N/A	N/A
**Pedram, 2013, Canada** [51]	Cross sectional	*n* = 652	Citizens of Canadian provinces Newfoundland and Labrador	63.7% female. Age: 44.3 ± 12.9 years (range 20–90). Age female 45.1 ± 12.9, male 42.9 ± 12.8 (*p* < 0.05). BMI: 27.4 ± 5.4 kg/m^2^ (range 17.05–54.2). Waist hip ratio 0.9 ± 0.08 (range 0.68–1.62). BMI female 26.8 ± 5.7, male 28.5 ± 4.6 (*p* < 0.05). Underweight/normal weight BMI 38.2%, OW/OB BMI 61.8%.	Investigation of food addiction in Canadian province and relationships with gender and weight status. 12 h fast prior to measurements	Anthropometrics; Body composition; Food frequency questionnaire (macronutrient intake); Physical activity	YFAS. No information re completion.	D + SS	N/A	N/A
**Gearhardt, 2012, USA** [27]	Cross sectional	*n* = 81	OB individuals who “eat out of control” and are seeking weight treatment	70.1% female Age: 47.47 ± 8.43 years (range 28–64) BMI: 40.58 ± 6.63 kg/m^2^. 79.3% Caucasian 82.6% had some college education.	Eating disorder confirmed by interview followed by battery of self-report questionnaires.	Anthropometrics; Eating Disorder Examination; BDI; Difficulties in Emotion Regulation Scale; Rosenberg Self Esteem Scale.	YFAS. No information re completion	D + SS	N/A	N/A
**Kromann, 2012, Denmark** [52]	Case report	*n* = 1	Female who believed she was addicted to cola.	40 year old female. Heavy smoker, depression, metabolic syndrome. Weight: 72.9 kg. FBG: 5.9 mmol/L, HDL: 1.17 mmol/L, triglycerides 0.75 mmol/L.	Patient used cola to boost energy (3 L daily). Offered cognitive therapy for depression. Reduced cola consumption to 200 mL.	Major Depression Inventory; Hamilton Depression Scale; Young Mania Rating Scale; Global Assessment of Functioning; Waist circumference; blood pressure; Fasting blood glucose; HDL; triglycerides.	YFAS Danish version	Points	6 months	N/A
**Meule 2012, 2012, Germany** [53,54,55]	Cross sectional	*n* = 617 completed all surveys. 1 excluded. Retest completed by *n* = 197	Students at German universities	38.2% survey completion rate 75.8% female Age: 24.5 ± 4.0 years. BMI: 22.3 ± 3.3 g/m^2^. 89% students 95.5% German citizens. 39.1% Non-dieters, 14.6% successful dieters, 22.7% unsuccessful dieters. Sig greater BMI in unsuccessful dieters than successful dieters and non-dieters (*p* < 0.001).	Validation of the German version of Food Cravings Questionnaire. Investigation of the relationships between food addiction and craving.	BMI; FCQ; Restraint scale subscale concern for dieting (RS-CD); Perceived self-regulatory success in dieting (PSRS); Flexible and rigid control of eating behavior; Eating disorder examination questionnaire (EDE-Q); Mannheimer Craving Scale (MaCS); BIS/BAS; PANAS.	YFAS. Online survey.	SS	N/A	N/A
**Meule, 2012, Germany** [56]	Cross sectional	*n* = 96	Individuals attending first bariatric surgery consultation	65.6% female Age: 39.92 ± 11.51 years BMI: 50.64 ± 8.99 (range 34.89–73.44 kg/m^2^). 91.7% had BMI ≥ 40 kg/m^2^.	Investigation of food addiction of individuals attending first bariatric consultation	-	German YFAS. Completed on day of bariatric consultation	D + SS	N/A	N/A
**Davis, 2011, Canada** [57]	Cross sectional	*n* = 72	Non clinical sample of OW/OB adults	68.1% female Age: Food addicts 35.3 years, non addicts 33.0 years. BMI food addicts 37.5 kg/m^2^, non addicts 38.8 kg/m^2^. % female food addicts 72.2%, non addicts 66.7%.	Validation of YFAs in non-clinical OW/OB individuals.	Eating Disorder Examination; BDI; Wender Utah rating scale for ADHD symptoms; BIS; Delay of gratification task; Eyesnck personality questionnaire revised; PFS; DEBQ (emotional and external eating); FCQ-T; Demographics	YFAS. Completed at home	D + SS	N/A	N/A
**Gearhardt, USA, 2011** [11]	Cross sectional	*n* = 48. *n* = 9 excluded	Recruited from a weight loss and maintenance program	100% female Age: 20.8 ± 1.31 years BMI: 28.0 ± 3.0 kg/m^2^ (range 23.8–39.2).	Neural responses of OB *vs.* normal weight individuals to milkshake.	Anthropometrics; fMRI	YFAS. No information re completion	SS	N/A	N/A
**Gearhardt, 2009, USA** [26]	Cross sectional	*n* = 353	Undergraduate students	Response rate 24.5%. 64.2% female Age: 20.11 ± 1.38 years. BMI: 22.58 ± 3.18 kg/m^2^. 73.5% HWR, 18.7% OW, 4.7% underweight, 2.7% OB. 72.5% Caucasian.	Initial validation study of YFAS.	BES; BIS/BAS; Eating Troubles Module; Emotional eating scale; Rutgers alcohol problem index; Daily drinking questionnaire.	YFAS. Part of larger health behaviors online survey.	D + SS	N/A	N/A

BDI = Beck Depression Inventory, BED = binge eating disorder, BIS/BAS = Behavioral Inhibition and Behavioral Activation Scale, BN = bulimia nervosa, D = Diagnosis, DEBQ = Dutch Eating Behavior Questionnaire, FCQ = Food Craving Questionnaire, HW = Healthy Weight, N/A = Non-applicable, NHS = Nurses Health Study, OB = Obese, OW = Overweight, PEMS = Palatable Eating Motives Scale, PFS = Power of Food Scale, RYGB = Roux-en-Y gastric bypass surgery, SS = symptom score.

**Table 2:** Quality assessment of the studies reviewed studies.

Author, year	Was a random or pseudo random sample used?	Was the inclusion criteria clearly defined?	Were confounding factors identified and control strategies stated?	Were outcomes assessed using objective criteria	Was there sufficient description of the groups?	Was follow up carried out over a sufficient time period?	Were the outcomes of people who withdrew described and included in analysis?	Were outcomes measured in a reliable way?	Was appropriate statistical analysis used?	Quality score
BRUNAULT 2014 [32]	UC	N	UC	Y	UC	N/A	UC	Y	Y	1
BURGESS 2014 [33]	Y	Y	Y	Y	Y	N/A	UC	Y	Y	6
DAVIS 2014 [34]	N	Y	Y	Y	Y	Y	UC	Y	Y	6
FLINT 2014 [35]	Y	Y	Y	Y	Y	N/A	Y	Y	Y	8
GEARHARDT 2014 [36]	Y	Y	Y	Y	Y	N/A	UC	Y	Y	6
IMPERATORI 2014 [37]	N	Y	Y	Y	UC	N/A	UC	Y	Y	3
LENT 2014 [38]	N	N	Y	Y	Y	N/A	Y	Y	Y	6
MEULE 2014 [40]	Y	Y	UC	Y	Y	N/A	Y	Y	Y	6
MEULE 2014 [41]	Y	Y	Y	Y	N	N/A	UC	Y	Y	5
MURPHY 2014 [43]	Y	N	Y	Y	N/A	N/A	UC	Y	Y	4
PEPINO 2014 [44]	N	Y	UC	Y	Y	Y	N	Y	Y	5
BURMEISTER 2013 [45]	Y	Y	UC	Y	N/A	Y	Y	Y	Y	6
CLARK 2013 [46]	UC	N	UC	Y	N/A	N/A	Y	Y	Y	2
DAVIS 2013 [47]	Y	Y	Y	Y	Y	N/A	UC	Y	Y	6
EICHEN 2013 [39]	N	UC	Y	Y	Y	N/A	UC	Y	Y	3
GEARHARDT 2013 [48]	Y	N	Y	Y	N/A	N/A	N	Y	Y	5
GEARHARDT 2013 [49]	Y	Y	UC	Y	Y	N/A	N	Y	Y	5
MASON 2013 [50]	Y	N	Y	Y	Y	N/A	UC	Y	Y	5
PEDRAM 2013 [51]	Y	Y	Y	Y	Y	N/A	UC	Y	Y	6
GEARHARDT 2012 [27]	Y	N	UC	Y	Y	N/A	UC	Y	Y	3
MEULE 2012 [56]	N	N	UC	Y	N/A	N/A	UC	Y	Y	1
MEULE 2012 [42]	Y	Y	UC	Y	Y	N/A	UC	Y	Y	4
MEULE 2012 [53]	Y	N	UC	Y	Y	N/A	N	Y	Y	4
MEULE 2012 [54]	Y	N	Y	Y	Y	N/A	N	Y	Y	6
DAVIS 2011 [57]	Y	Y	UC	Y	Y	N/A	UC	Y	UC	2
GEARHARDT 2011 [11]	UC	Y	Y	Y	N	N/A	N	Y	Y	4
GEARHARDT 2009 [26]	Y	N	UC	Y	N/A	N/A	N	Y	Y	3

A total of 196,211 participants were examined across reviewed studies ranging from one to 134,175 participants. Participants were predominantly female, with six studies investigating females exclusively [11,35,40,41,42,50,52] and an additional nine studies investigating a population with >70% female participants [27,33,36,37,38,39,43,44,49,53,54]. Age of included participants ranged from four to ninety years. Fourteen studies included older adults aged >35 years [27,35,37,38,39,44,45,46,49,50,51,52,56], ten studied younger adults aged from 18–35 years [11,26,32,33,34,36,40,41,42,43,47,53,54,57], and one studied children and adolescents <18 years [48]. Seven studies investigated a healthy weight population (18.5–25 kg/m^2^) [26,32,35,40,41,42,43], four studied an overweight population (25–30 kg/m^2^) [11,33,36,51], and ten studied an obese population (>30 kg/m^2^ [27,34,37,38,39,44,45,46,47,49,56,57]. Four studies did not specify the BMI or weight category of participants [46,48,50,52]. However, the study conducted by Clark *et al.* [46] investigated bariatric surgery patients who, according to Clinical Practice Guidelines, would have been likely to have a BMI ≥35 kg/m^2^ [58].

The standard YFAS comprised of 25 self-report questions was used in 23 studies. Two studies used the modified YFAS (m-YFAS) which consisted of nine core questions including one item for each symptom plus two items for clinical impairment and distress [35,50]. The YFAS modified for children (YFAS-C) was used in one study and consisted of 25 questions which were changed to age appropriate activities and a lower reading level [48]. Five of the reviewed studies were completed online [26,32,35,46,53,54]. Four studies specifically noted that the YFAS was translated into a language other than English (Italian, German and French) [32,37,40,54], and one study changed the reporting period of twelve months used in the original YFAS to the previous one month [38,39] to give a more proximal indication of YFAS outcomes following an intervention. Fifteen studies investigated both the YFAS diagnosis and symptom score [26,27,32,36,37,38,39,40,43,44,46,48,49,51,56,57], five used the symptom score exclusively [11,33,41,42,45,53,54] and four used the diagnosis exclusively [34,35,47,50]. Two studies grouped participants as “high” or “low” FA based on the number of YFAS symptoms endorsed [11,41,42]. One of these studies used this method of categorization as <5% of participants met the diagnostic cut offs [11] while the second study gave no rationale for this method of scoring [42]. One study used a numerical point score with no description from the authors regarding the meaning of this score [52].

### 3.1. Prevalence of FA Diagnosis

Twenty-three studies reported the prevalence of FA diagnosis. As shown in Table 3, the proportion of the population samples meeting the diagnostic criteria for FA ranged from 5.4% [51] to 56.8% [27]. Twenty studies reported the mean prevalence of FA for the whole sample and were meta-analyzed (Table 4). Meta-analysis identified significant heterogeneity in the included studies and thus the random effects model is reported. Meta-analysis revealed that this review was not subject to publication bias.

**Table 3:** Results of included studies using the Yale Food Addiction Scale to assess food addiction.

Author, year, country	Prevalence of FA by diagnosis	YFAS symptoms	YFAS outcomes in association with other variables	Conclusions	Limitations
**Brunault, 2014, France** [32]	FA diagnosis: 8.7%	Median symptoms: 1, mean YFAS symptoms: 1.9 ± 1.4 (95% CI 1.8–2.0).	DIAGNOSIS: Diagnosis of food addiction associated with higher binge eating scores using BITE and BES (*p* < 0.001). SYMPTOM SCORE: YFAS symptom score was significantly correlated with the binge eating scores using BITE (*p* = 0.59, *p* < 0.001), and BES (*p* = 0.58 *p* < 0.001).	French version of the YFAs is a psychosometrically sound tool to assess food addiction.	No sex reported
**Burgess, 2014, USA** [33]	-	-	SYMPTOM SCORE: Sig associations between YFAS and all subscales of the PEMS (*p* < 0.001).	Provided preliminary validation of the PEMS. PEMS accounted for unique variability in predicting BMI.	Little detail regarding FA
**Davis, 2014, Canada** [34]	FA diagnosis: 16.9%	-	DIAGNOSIS: Food addiction group reported higher food cravings and appetite ratings (*p* < 0.0001). Methylphenidate compared to placebo reduced snack food intake in the non-food addiction group (*p* < 0.0001) but did not significantly change the snack food consumption of food addicts.	Methylphenidate did not change the snack food consumption or appear to reduce the appetite of food addicts.	Small sample, limited range of snack foods
**Flint, 2014, USA** [35]	FA diagnosis whole sample 5.8%. FA diagnosis NHS II 8.4% NHS 2.7%.	Most common symptoms: NHS: Consumption despite significant problems 15.6%; Eating same amount of food does not produce same feelings 18.4%. NHS II: Consumption despite significant problems 22.3%; Cutting down foods 22.9%; Consume foods despite not being hungry 17.8%	Women with FA more likely to be OW. Reduction in FA prevalence with increasing age (45–61 years: 7.4%–9.4%, 62–70 years: 2%–3%). Increasing prevalence of FA with higher BMI (BMI < 25 kg/m^2^: 0.4%–2.8%: BMI 25–30 kg/m^2^: 1.6%–8.4%, >30 kg/m^2^: 6.1%–24.7%). Proportion of FA diagnosis higher in white women. FA less common in more physically active women. FA positively associated with hypercholesterolaemia, former smoking and depression. Modified YFAS had similar internal consistency, convergent validity as the original YFAS.	FA prevalence as assessed by the modified YFAS ranged from 1%–9% in middle aged and older women. FA was negatively associated with age and positively associated with BMI	No measures of eating disorders, female only, limited generalizability
**Gearhardt, 2014, USA** [36]	FA diagnosis whole sample: 25.7% FA diagnosis BN 83.6%, FA diagnosis BED 47.2%	Mean symptoms: 3.05 ± 2.0	DIAGNOSIS: FA associated with higher current BMI, higher lifetime BMI, earlier age of first dieting, current dieting, and weight cycling (*p* < 0.001). Food addiction significantly associated with all disordered eating variables including binge eating behaviors (*p* < 0.001). Higher prevalence of FA diagnosis in BN than BED (*p* < 0.001). FA alone had a higher BMI than all other groups (*p* < 0.001). BED + FA and BN + FA reported more dieting than FA or BED/BN alone (*p* < 0.001). BN + FA had highest subjective binge episodes compared to other groups followed by BED + FA (*p* < 0.001). Sig higher restraint in FA (*p* < 0.001).	FA prevalence higher in BN than BED and associated with BMI and eating pathology.	Self-reported measures used to identify eating disorders, predominantly female, higher rates of eating disorders than expected
**Imperatori, 2014, Italy** [37]	FA diagnosis: 33.9%.	Mean symptoms 2.68 ± 1.89	28.9% food addicts met criteria for BED compared to 4.1% of non-food addicts. YFAS had strong positive correlation with BES (*r* = 0.78, *p* = 0.0045), and moderate positive correlations with all the psychopathology criteria (*p* = 0.0045) except phobic anxiety. More severe FA was associated with more severe psychopathology when mediated by BES (*p* < 0.001).	FA associated with binge eating severity and psychopathology. Relationship of FA and psychopathology completely mediated by BES.	Small sample size, self-reported measures.
**Eichen, USA, 2013. Lent 2014 USA** [38,39]	Baseline FA diagnosis: 15.2%.	Mean symptoms: 2.57 ± 1.67. 45% reported ≥3 symptoms. Most common symptoms: Inability to cut down or stop eating (96.1%); continued use despite consequences (44.4%); tolerance (36%).	DIAGNOSIS: Food addicts had higher BDI scores (*p* < 0.001). No effect of FA status on weight loss when controlling for treatment, gender and baseline weight.SYMPTOM SCORE: Higher number of symptoms reported by females (*p* = 0.04) and African Americans (*p* = 0.002). Negative correlation between age and number of symptoms (*r* = −0.284 *p* < 0.001). Positive relationship between symptom count and BDI scores (*r* = 0.48, *p* < 0.001). YFAS symptom score did not account for unique variance in weight change. FA status and symptom count not associated with attrition.	15% of OW and OB individuals were found to meet the criteria for FA. Baseline FA status did not predict weight loss success or attrition.	Only assess eating behaviors in previous month, predominantly female, short term weight loss, limited generalizability.
**Meule 2014 Germany** [40]	FA diagnosis: 100% current BN, 30% remitted BN, 0% control group (*p* < 0.001).	Mean symptoms: current BN 6.27 ± 1.04, remitted BN 3.95 ± 1.79, control 0.86 ± 0.90 (*p* < 0.001). Most common symptoms: persistent desire or unsuccessful attempts to cut down, giving up activities and withdrawal symptoms.	DIAGNOSIS: Food addiction group had fewer years of education (*p* = 0.02) and a lower BMI (*p* = 0.08) than the no FA group. Food addiction group had higher eating disorder psychopathology (*p* < 0.001). Food addicted had higher depression scores (*p* < 0.001) and binge eating behavior (*p* < 0.001). SYMPTOM SCORE: YFAS symptom score positively correlated with all measures of eating disorder psychopathology (total *r* = 0.82, *p* < 0.001) and negatively correlated with BMI (*r* = −0.19, *p* = 0.05). Higher number FA symptoms related to depression (*r* = 0.60, *p* < 0.001) and binge eating behaviors (*r* = 0.65, *p* < 0.001).	BN symptomology strongly associated with addictive eating as measured by the YFAS and attenuates the positive relationship between FA and BMI.	Female only, self-report, bulimia not assessed using diagnostic interviews
**Meule 2014 2012, Germany** [41,42]	Low FA group 60%, High FA group 40%.	Mean symptoms: Low FA group: 0.83 ± 0.38 (range 0–1), High FA group: 2.65 ± 0.75 (range 2–4).	HIGH VS LOW FA: High FA group were younger (*p* < 0.05) and had higher levels of self-reported attentional impulsivity (*p* < 0.05). High FA had faster reaction times to food images compared to neutral cues (*p* < 0.01) in XY task. SYMPTOM SCORE: FA symptoms positively correlated with BMI (*r* = 0.42, *p* < 0.01), self-reported impulsivity (*r* = 0.344, *p* < 0.05) and depressive symptoms (*r* = 0.29, *p* < 0.05). FA symptoms not correlated with task performance or the FCQ-S. FA symptoms did not differ between dieters and non-dieters.	FA symptoms related to accelerated responses to high calorie food cues, BMI and heightened self-reported attentional impulsivity. Response time was slower to foods than neutral cues, reduced inhibitory control when shown food images which was predictive of food craving.	Small sample size, female only, used high and low FA not diagnostic criteria, did not exclude moderate scores
**Murphy 2014 USA** [43]	FA diagnosis: 24%.	Mean symptoms: 1.80 ± 1.39.	SYMPTOM SCORE: BMI and FA positively correlated (*r* = 0.18, *p* < 0.01). Impulsivity measures including negative urgency (*p* < 0.01) and perseverance (*p* < 0.05) significant predictors of number of symptoms endorsed.	FA symptoms associated with BMI and impulsivity.	Few people met criteria for FA, predominantly female, limited respresentativeness
**Pepino, 2014, USA** [44]	FA diagnosis pre surgery: 32% FA diagnosis post-surgery: 2%. *p* < 0.05 93% changed FA diagnosis	Food addicts had more symptoms pre weight loss compared to non-food addicts (5.0 ± 1.0, 2.0 ± 0.5 respectively, *p* < 0.0001). Surgery reduced mean number of symptoms (*p* < 0.0001).	Food addicts greater cravings than non addicts pre surgery. Food addicts had greater reductions in cravings post-surgery than non-food addicts (*p* < 0.05). Food addicts had greater craving of fast foods and starchy foods pre surgery (*p* < 0.05) and greater cravings of starchy foods after surgery (*p* = 0.009). Surgical weight loss reduced emotional and external eating in all subjects, restrained eating behavior decreased in food addicts only (*p* < 0.05 Cohen’s *d* ≥ 0.80).	Weight loss following bariatric surgery is an effective treatment for food addiction and abnormal eating behaviors associated with food addiction.	Did not assess BED, dietary counselling may influence eating behaviors, limited generalizability
**Burmeister, 2013, USA** [45]	FA diagnosis: 19.6%. FA diagnosis males 19.3%, female 14.0%.	-	SYMPTOM SCORES: Greater FA scores more likely to have psychological distress and depression (*r* = 0.50, *p* <0.01). Higher FA scores positively related to binge eating behaviors, emotional eating, difficulty controlling eating, weight bias internalisation, fear of fat and body shame (*p* < 0.01). High YFAS scores negatively related to percent weight loss at week 7 (*r* = −0.24, *p* = 0.04).	Number of FA symptoms related to negative attitudes about being OW/OB. Number of FA symptoms endorsed was negatively correlated with weight loss.	Small sample size, predominantly female- limited generalizability, short follow up
**Clark, 2013, USA** [46]	FA diagnosis 53.7% pre surgery.	-	DIAGNOSIS: Sig relationship between YFAS diagnosis and emotional eating, binge eating and symptoms of eating disorders (*p* < 0.05). Food addicts had a non-significant poorer weight loss (27% *vs*. 32%). SYMPTOM SCORE: Sig relationship between YFAS symptoms and emotional eating and binge eating scale (*p* < 0.05). When controlling for eating disorders and emotional eating, YFAS explained 6% (*p* = 0.014) of variance in binge eating scores.	YFAS contributed unique variability in predicting binge eating.	Small sample
**Davis, 2013, Canada** [47]	FA diagnosis 17.5%. FA diagnosis females 13.3%, males 4.2%	-	DIAGNOSIS: Food addicts had higher MLGP scores than non-food addicts (*p* = 0.023). Higher hedonic eating, binge eating, emotional eating, food craving and sweet snacking scores in food addicts compared to non-food addicts (*p* < 0.0001).	Food addicts had higher MLGP scores, food craving, binge eating, emotional eating compared to non-addicted.	Few individuals met FA diagnostic criteria.
**Gearhardt, 2013, USA** [48]	FA diagnosis: 7.2%	Median number symptoms 2 ± 1.81. ≥3 symptoms reported by 38.6%. Given up activities 38.7%; loss of control 29.3%; tolerance 24.7%; inability to cut down 65.3%; withdrawal 18.9%; large amount of time spent 25.7%; clinically sig impairment or distress 9.6%	-	Preliminary evidence that the YFAS-C is a valid and reliable tool to operationalise FA in children.	Children’s dietary preferences often dictated by parents.
**Gearhardt, 2013, USA** [49]	FA Diagnosis: 41.5%.	Mean symptoms: 4.33 ± 1.81. Consumed more than planned 58.9%; unable to cut down 100%; much time spent 67.4%; activities given up 38.9%; use despite consequences 60.6%; tolerance 61.7%; withdrawal 44.2% impairment or distress 42.6%	DIAGNOSIS: FA diagnosis sig associated with earlier age of being OW (*p* = 0.014). SYMPTOM SCORE: YFAS positively correlated with negative affect and emotional dysregulation, and negatively correlated with self-esteem (*p* = 0.01). YFAS scores positively correlated with frequency of binge eating, eating concern, and weight concern (*p* < 0.05). YFAS scores associated with earlier age of being OW (*r*^2^ = −0.24, *p* = 0.015), age of dieting onset (*r*^2^ = −0.21, *p* = 0.037). YFAS sig accounted for 11% of unique variance in BED (*p* < 0.001).	FA rates not different in different ethnic groups. FA may represent a more severe presentation of BED.	Limited generalizability, retrospective self-reported measures
**Mason, 2013, USA** [50]	FA diagnosis: 8.2%	Symptoms endorsed by ≥70% of participants with FA except tolerance and withdrawal.	DIAGNOSIS: Greater BMI in women meeting FA criteria (≥2/3 food addicts classified as obese compared to ¼ non-food addicts). Risk ratios for FA increased with severity of physical abuse up to RR 1.92 (CI 1.76–2.09). Risk ratios for FA increased with severity of sexual abuse up to RR 1.87 (CI 1.69–2.05). Sexual and physical abuse combined increased risk for FA up to RR 2.40 (CI 2.16–2.67). Longer duration of abuse conferred greater FA risk.	Dose response associations between physical and sexual abuse and likelihood of FA.	Unable to determine time course of FA and abuse. Female only, predominantly white-limits generalizability.
**Pedram, 2013, Canada** [51]	FA diagnosis: 5.4%. FA diagnosis female 6.7%, male 3.0%. FA diagnosis in BMI UW/ HW: 1.6%, OW/OB 7.7%. FA diagnosis percent body fat UW/HW 2.9%, OW/OB 6.8%.	-	DIAGNOSIS: Risk of FA higher in females than males (RR = 2.28, *p* = 0.046). OW/ OB women had higher risk of FA compared to OW/OB men (RR = 3.50, *p* = 0.002). Proportion of FA significantly increased with increasing adiposity (UW/HW RR = 0.21, *p* < 0.001, OW/OB RR = 0.42, *p* = 0.03). Food addicts had higher BMI (difference +4.6 kg/m^2^, *p* < 0.001), weight (difference +11.7 kg, *p* <0.001), body fat (difference +8.2%, *p* < 0.001), trunk fat (difference +8.5%, *p* < 0.001). Food addicts reported greater intake of fat (difference +2.3%, *p* = 0.04) and protein (difference +1.1%, *p* = 0.04). SYMPTOM SCORE: FA symptoms correlated with BMI (*r* = 0.36, *p* < 0.001), weight (*r* = 0.35, *p* < 0.001), waist hip ratio (*r* = 0.15, *p* < 0.001), percent body fat (*r* = 0.31, *p* < 0.001), and trunk fat (*r* = 0.32, *p* < 0.001).	Prevalence of FA in the Canadian province was 5.4%. Females at greater risk of FA and FA related to obesity.	Predominantly female
**Gearhardt, 2012, USA** [27]	FA diagnosis 56.8%.	Mean symptoms 4.56 ± 1.9. 57.1% who did not meet food addiction criteria endorsed ≥3 symptoms. Consumed more than planned 57.3%; unable to cut down or stop 1.2%; great deal of time spent 32.9%; important activities given up 53.7%; use despite consequences 24.7%; tolerance 43.2%; withdrawal 45.1%; impairment or distress 38.3%.	Food addiction related to mood disorder diagnoses (*p* = 0.01) specifically major depressive disorder (*p* = 0.06). YFAS symptoms positively correlated with BDI, and negatively correlated with self-esteem (*p* < 0.01). YFAS positively correlated with restraint and frequency of binge eating, (*p* < 0.01), and eating disorder psychopathology (*p* < 0.05). YFAS scores accounted for 6.3% unique variance in binge eating scores (*p* = 0.28).	BED classified as food addicts appear associated with mood disorders, lower self-esteem and eating disorder psychopathology in patients with BED.	Non control group, predominantly female.
**Kromann, 2012, Denmark** [52]	-	Scored 40 points on the YFAS at baseline. Following reduction in cola intake YFAS score was 0.	Patient fulfilled all DSM criteria for substance addiction with respect to cola. Abstinence from cola caused obsession and craving for cola. Reduction in cola consumption increased concentration, feelings of wellness, reduction in psychopharmacological medication. Reduction in weight, and no longer fulfilled criteria for metabolic syndrome following reduction in cola consumption.	Overconsumption of caffeinated drink may have caused or accentuated mental health problems.	Singular case study, not generalizable
**Meule 2012, 2012, Germany** [53,54,55]	FA diagnosis 7.8%.	-	DIAGNOSIS: Food addicts had higher total FCQ-T total and FCQ subscales scores score (*p* < 0.001) except for positive reinforcement. SYMPTOM SCORE: Positive reinforcement negatively predicted FA symptoms (*p* < 0.001). Negative relationship between FA symptoms and anticipation of positive reinforcement in low FA scores (beta = −0.32, *p* < 0.001), but was weak for those with high food cravings (beta = −0.12, *p* < 0.01). FA symptoms positively correlated with FCQ-T scores (*r* = 0.50, *p* < 0.001).	German FCQ is a reliable and valid measure of food cravings. Individuals with high food cravings and low anticipation of positive reinforcement displayed most FA symptoms.	Predominantly female, HW non clinical sample- limits generalizability
**Meule, 2012, Germany** [56]	FA diagnosis 41.7%.	Mean symptoms 3.42 ± 1.74. Persistent desire 94.8%; continual eating despite problems 75%; tolerance 54.2%; clinically sig impairment or distress 47.9%; consume larger amounts over longer period than intended 31.3%; spending much time obtaining or recovering 30.2%; giving up activities 29.2%; withdrawal 27.1%	Items related to persistent desire or repeated unsuccessful attempts to cut down do not differentiate well between food addicts and non addicts. Questions regarding continual consumption and activities given up endorsed by greater number of respondents in BED current study than non-clinical samples in other studies. 75% respondents- much higher than non-clinical samples.	Factor structure of the YFAS could be confirmed in a sample of OB individuals seeking bariatric surgery. Some items have poor item based on population studied.	No assessment of mental disorders, small sample size
**Davis, 2011, USA** [57]	FA diagnosis 25%. FA diagnosis female 18.1%, male 6.9%.	-	DIAGNOSIS: FA associated with higher BED (*p* < 0.0001) and depression (*p* = 0.018). Food addicts displayed greater impulsivity (*p* < 0.0001), addictive traits (*p* = 0.003) and delay of discounting (*p* = 0.035). Food addicts reported greater binge eating traits, hedonic eating, emotional eating, food cravings, and snacking on sweets (*p* < 0.0001).SYMPTOM SCORE: Addictive traits, hedonic eating, snacking on sweets and binge eating scores accounted for 56% variance in YFAS symptom scores.	Strong relationships between food and substance abuse in OB. Validation of the YFAS’s ability to identify individuals with addictive like eating behaviors.	Limited number of food addicts. Limited power. Small sample size precluded gender analysis
**Gearhardt, 2011, USA** [11]	High FA 38.5%, Low FA 28.2%. Only *n* = 2 (5.1%) reported clinical impairment.	Mean symptom score for High FA 3.60 ± 0.63, All Low FA only had 1 symptom.	HIGH VS LOW FA: High FA had greater brain activation than Low FA in the dorsolateral prefrontal cortex (*p* < 0.007), caudate (*p* < 0.004). SYMPTOM SCORE: YFAS correlated with emotional eating (*p* < 0.03), and external eating (*p* < 0.02). Positive correlations YFAS scores and brain activity in the anterior cingulate cortex (*p* < 0.001), medial orbitofrontal cortex (*p* < 0.004) and amygdala (*p* < 0.007).	FA scores are associated with activation of reward regions of the brain. Compulsive food consumption may be driven by the reward anticipation.	Few reported clinical impairment-symptom count used not diagnosis, small sample size.
**Gearhardt, 2009, USA** [26]	FA diagnosis: 11.4%	Median symptoms = 1.	DIAGNOSIS: FA diagnosis accounted for 5.8% unique variance in binge eating scores. SYMPTOM SCORE: YFAS symptom count accounted for 14.8% unique variance in binge eating	YFAS is a psychosometrically sound tool to assess FA	Limited response rate, limited representativeness. Self-reported anthropometrics

BDI= Beck Depression Inventory, FA= Food addiction, Healthy weight= HW, Overweight= OW, Obese= OB, Underweight= UW, RR= relative risk, Sig = significant.

**Table 4:** Meta-analysis results of food addiction prevalence by gender, weight status, age, and disordered eating status. Random effects model is reported.

Population group (*n* = number of studies)	Weighted mean prevalence of food addiction diagnosis (%)	95% Confidence interval (%)	Range of prevalence across all studies (%)
Total (*n* = 20)	19.9	16.3, 24.0	5.4%–56.8%
**Gender**			
Male (*n* = 4)	6.4	2.4, 16.0	2.4–16.0
Female (*n* = 6)	12.2	6.4, 21.4	6.7–21.4
**Weight status**			
Healthy weight (*n* = 5) ^a^	11.1	6.3, 18.9	1.6–24.0
Overweight/obese (*n* = 13) ^b^	24.9	14.2, 40.1	7.7–56.8
**Age group**			
Adults < 35 years (*n* = 9)	17.0	11.8, 23.9	7.8–25.0
Adults > 35 years (*n* = 11)	22.2	17.7, 27.5	5.4–56.8
**Disordered eating**			
No clinically diagnosed disordered eating (*n* = 16)	16.2	13.4, 19.4	5.4–25.0
Clinically diagnosed disordered eating (*n* = 4) ^c^	57.6	35.3, 77.8	26.1–62.0

^a^ Healthy weight BMI < 25 kg/m^2^; ^b^ Overweight/obese BMI ≥ 25 kg/m^2^; ^c^ Includes binge eating disorder and bulimia nervosa samples.

**Figure 2:**
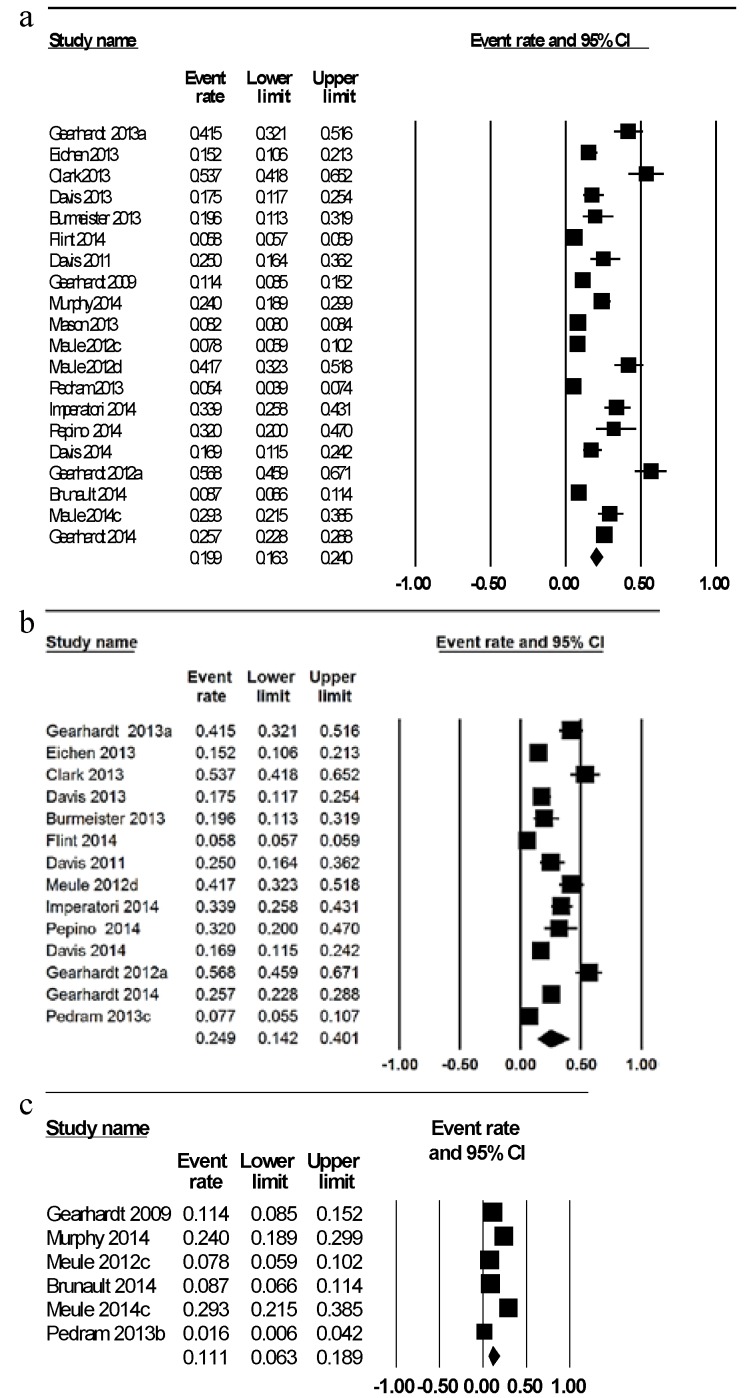
(**a**) Meta-analysis of Yale Food Addiction Scale Diagnosis for all studies; (**b**) Meta-analysis of Yale Food Addiction Scale Diagnosis for overweight/obese samples; (**c**) Meta-analysis of Yale Food Addiction Scale Diagnosis for healthy weight samples.

The weighted mean prevalence of FA across all studies was 19.9% (Figure 2a) [26,27,32,34,35,36,37,39,40,43,44,45,46,47,49,50,51,53,56,57]. Prevalence of FA diagnosis was meta-analyzed by sex with a higher mean prevalence of FA in six samples of females exclusively of 12.2% [35,40,45,47,51,57] compared to 6.4% in four samples of males [45,47,51,57]. When meta-analyzed by BMI category, the mean prevalence of FA was considerably greater at 24.9% in fourteen studies investigating overweight/obese individuals (Figure 2b) [27,34,35,36,37,38,39,44,45,46,47,49,51,56,57] compared to 11.1% in six studies of healthy weight individuals (Figure 2c) [26,28,32,43,51,53]. The mean FA prevalence was lower in nine samples of adults younger than 35 years of age at 17.0% [26,32,34,36,40,43,47,53,57] compared to 22.2% in eleven samples of adults aged over 35 years [27,35,37,39,44,45,46,49,50,51,56]. However, in one study reporting the outcomes of adults aged 62–88 years as well as adults aged 42–64 years, the prevalence of FA diagnosis was lower in the older age group (2.7% and 8.4% respectively) [35]. In the single study of children and adolescents <18 years the prevalence of FA was 7.2% [48].

When categorized by disordered eating status the mean prevalence of FA was 57.6% in four samples with a clinically diagnosed eating disorder [27,36,40,49] and 16.2% in sixteen samples of individuals with no clinical diagnosis of disordered eating. Prevalence of FA diagnosis in two studies of individuals with diagnosed BED was 41.5% and 56.8% [27,49]. The prevalence of FA diagnosis in individuals with a current diagnosis of bulimia nervosa was 83.6% and 100%, while 30% of individuals with a history of bulimia nervosa met the diagnostic criteria for FA [36,40]. In the single study assessing FA at two time points, baseline and nine months, prevalence of FA diagnosis was found to reduce from 32% to 2% following bariatric surgery which following a mean weight loss of 20% original body mass [44].

### 3.2. Prevalence of FA Symptoms

Sixteen studies reported the total number or specific symptoms endorsed by participants. Eight studies reported the mean number of symptoms for the whole study sample and were meta-analyzed [27,32,36,37,38,39,42,43,49,56]. The weighted mean number of symptoms reported was 2.8 ± 0.4 (95% CI 2.0, 3.5) and ranged from 1.8 [43] to 4.6 [27] symptoms out of a possible total score of seven. Clinical samples (six studies) endorsed a mean 4.0 ± 0.5 symptoms (95% CI 3.1, 4.9) [27,37,38,39,40,49,56] while non-clinical samples (five studies) endorsed a mean 1.7 ± 0.4 symptoms (95% CI 0.9, 2.5) [32,36,40,43]. Seven studies reported the frequencies of specific FA criteria and in five of these studies the most common symptom reported was “*the persistent desire or unsuccessful attempts to cut down foods*” [39,40,48,49,56]. Other commonly reported symptoms ranged based on the population studied.

### 3.3. Relationship of YFAS Outcomes with Other Variables

Across the reviewed studies, YFAS diagnosis and symptom score were associated with a variety of anthropometric measures. Specifically, higher BMI’s were related to higher rates of FA diagnosis [35,36,50,51] and number of symptoms endorsed [41,42,43,51]. However, in one study of individuals with BN, FA diagnosis and higher symptom scores were associated with a significantly lower BMI [40]. Symptom score was positively correlated with other measures of adiposity including waist-to-hip ratio, percent body fat and trunk fat [51]. One study identified a relationship between YFAS symptom score and weight loss after a seven week behavioral weight loss intervention [45] while a second study found no relationship between weight change after a six month intervention and baseline YFAS outcomes [38].

In support of the results of the pooled meta-analysis, prevalence of FA diagnosis and number of symptoms reported decreased with increasing age [35,39] and females were found to have a higher prevalence of FA diagnosis and higher symptom scores [39,51]. Two studies identified ethnicity differences with one reporting higher FA scores in African Americans [39] and a second reporting prevalence of FA diagnosis to be higher in white females [35]. However, other studies identified no differences in FA prevalence based on ethnicity [36,49]. Diagnosis of FA was associated with health indicators including high cholesterol, smoking and decreased physical activity in one large scale epidemiological study [35].

Three studies examined relationships between the YFAS and foods or nutrients. Only one of these used a standardized dietary assessment method [51]. Individuals with a FA diagnosis were reported to have a significantly greater proportion of energy intake from fat (mean difference = +2.3%, *p* = 0.04) and protein (mean difference = +1.1%, *p* = 0.04) compared to individuals with no FA diagnosis [51] as measured by the Willett Food Frequency Questionnaire [59]. A case study of potential cola dependence demonstrated that YFAS scores reduced with a reduction in the amount of the cola consumed [52]. In addition, individuals classified as food addicted displayed greater pre bariatric surgery cravings of starchy foods and fast foods [44]. Interestingly, methylphenidate, a drug known to decrease appetite, did not reduce snack food consumption in individuals meeting the diagnostic criteria for FA [34]. One study used brain activity, measured by functional magnetic resonance imaging (fMRI), to assess neural responses to food cues and identified a positive correlation between YFAS symptom score and brain activity in a similar pattern to drug and alcohol addiction [11].

The YFAS was commonly assessed in combination with other tools including the Binge Eating Scale (six studies) [26,32,33,37,45,46], Eating Disorder Examination (six studies) [27,36,40,49,54,57], Food Craving Questionnaire (five studies) [34,41,42,47,53,54,57], Dutch Eating Behavior Questionnaire (five studies) [40,44,45,47,57], and the Beck Depression Inventory (four studies) [27,39,49,57]. Binge eating behaviors were higher in individuals meeting the diagnostic criteria for FA [32,36,37,40,46,47,57] and the YFAS diagnosis accounted for 5.8% unique variance in binge eating scores above and beyond other measures of eating pathology [26]. FA symptom scores were also positively associated with binge eating behaviors [27,32,37,40,45,46,49], with symptom scores accounting for 6%–14.8% unique variance in BED scores [26,46,49]. Diagnosis of FA and symptom score were positively associated with eating disorder psychopathology [27,36,37,40,46]. Higher depression scores were related to diagnosis of FA [27,35,39,40,57] and higher symptom scores [27,39,41,42,45]. Diagnosis of FA and symptom score were significantly positively related to a variety of eating behavior variables including emotional and external eating [11,45,46,47,57], food cravings [34,44,47,53,54,55,57], impulsivity [41,42], hedonic eating and snacking on sweets [47,57], In one study assessing FA at two time points, bariatric surgery reduced food cravings and restrained eating behavior in food addicts [44].

### 3.4. Comparison of “High” vs. “Low” FA

No standardized definition for “high” and “low” FA scores were used in the two studies describing YFAS outcomes using this approach. In one of these studies, 35.8% were classified as “high” FA if they endorsed ≥3 symptoms and 28.2% as “low” FA if they endorsed ≤1 symptom, with individuals with moderate FA scores excluded [11]. The second classified participants based on median split of symptom scores with 60% participants subsequently classified as “high FA” (2–4 symptoms) and 40% classified as “low FA” (≤1 symptom) [41,42]. In studies using high and low FA groups, the high FA group were significantly younger, had higher levels of attentional impulsivity, faster reaction times to food cues [43] and had greater brain activation to food cues compared to non-food addicts [11].

## 4. Discussion

This review aimed to systematically appraise studies that have used the YFAS to assess the presence of FA diagnosis or FA symptoms in a specified population. Using meta-analysis, the weighted mean prevalence of FA diagnosis in adult population samples was 19.9%. Meta-analysis indicated that FA prevalence was double that in overweight/obese population samples compared to those of a healthy BMI (24.9% and 11.1% respectively) and in females compared to males (12.2% and 6.4% respectively). FA prevalence was also higher in adults older than 35 years compared to adults younger than 35 years (22.2% and 17.0% respectively). Additionally, in populations with disordered eating, mean prevalence of FA was 57.6%, which was higher than that of individuals with no clinical diagnosis of disordered eating at 16.2%. The mean number of symptoms reported across studies was three out of a possible seven symptoms and the most common symptom reported in 70% of studies was “*persistent desire or unsuccessful attempts to cut down food intake*”. When meta-analyzed by clinical status, clinical populations endorsed more than double the number of symptoms compared to non-clinical populations (4.0 and 1.7 symptoms respectively). However, it should be noted that the population samples in the included studies were predominantly comprised of overweight/obese females recruited from clinical settings. Hence, the prevalence of YFAS FA diagnosis and the average symptom scores are likely higher compared to a nationally representative general population sample due to the characteristics of included participants.

It has been suggested that an addiction to food could act in a similar way to other substance addictions, with repeated exposures to pleasurable food diminishing the dopamine brain response [60,61]. This would lead to larger quantities of food consumed in order to feel satisfied, subsequently perpetuating overeating. This could help to explain why the meta-analysis conducted in this review identified that older adults displayed a higher prevalence of FA, with repeated exposures to a specific food over a person’s lifetime reducing the dopaminergic reward response. In contrast to this hypothesis, the study conducted by Flint *et al*., females aged over 62 years had a lower prevalence of FA diagnosis than a group of middle aged females 42–64 years [35]. A similar phenomenon has been noted in craving and consumption of alcohol, with reductions observed in older adulthood [62,63]. It has been postulated that this may be due to age-related neurodegenerative changes in dopaminergic release [62], and it could be possible that a similar event is occurring in FA. Further research exploring differences in FA status over a person’s lifetime is required to substantiate this theory.

Overconsumption and subsequent weight gain related to a blunted dopaminergic response could also provide a rationale for the finding that FA prevalence was higher in overweight/obese individuals. Of note, while YFAS diagnosis and symptom scores were positively related to anthropometric variables associated with adiposity in numerous studies reviewed, including a range of weight categories [35,36,51], other factors such as the presence of bulimia nervosa was found to attenuate this relationship [40]. Therefore, there remain limitations to equating obesity status with addictive-like eating and further research is required.

Meta-analysis also identified that females had a higher prevalence of FA compared to males, which may be attributable to gender related differences in hormonal profiles and/or dietary patterns [64,65]. Very few studies reported the diagnosis in males exclusively, hence the results of the meta-analysis should be interpreted with caution. While two studies identified relationships between FA symptoms and ethnicity, the specific ethnicity with highest FA prevalence differed between the studies [35,39]. These ethnic relationships may be influenced by the demographic composition of population samples. Further investigation in representative samples and controlling for potential confounding variables is required before the relationships between adiposity, gender and FA can be confirmed or refuted.

The majority of reviewed studies were cross sectional in design, assessing FA via the YFAS at one time point only. This precludes interpretation of cause and effect among variables. Only one study included in the review was classified as positive quality [35], which may be a result of the observational nature of the included studies. A single study tracked FA over time in the same population and assessed prevalence of FA before and nine months after gastric bypass surgery [44]. In this study diagnosis of FA was found to subside in thirteen of the fourteen participants classified as food addicted at baseline. This could provide some evidence that weight loss post bariatric surgery could reverse addictive-like eating behaviors, as assessed by the YFAS.

In contrast, studies of behavioral weight loss interventions reported disparate findings in the relationship between weight loss and YFAS outcomes. While one study found that YFAS scores at baseline predicted weight loss, a second longer term study found no relationship between FA status and weight loss success [38,45]. Although 30% of studies investigated FA in a population seeking or participating in weight loss, no studies conducting a behavioral weight loss intervention have reported YFAS outcomes at the conclusion of the intervention. Modifying the reporting period of the YFAS from the original twelve months to a shorter timeframe would be useful in combination with a behavioral weight loss intervention to determine if addictive food behaviors have changed over the discrete period of the therapy and at follow up.

Individuals with diagnosed eating disorders including BED and bulimia nervosa were shown to have a higher prevalence of FA [27,36,40,49], as assessed by the YFAS, compared to non-clinical population samples. Only two studies investigated FA in BED patients exclusively, despite a number of studies demonstrating a relationship between YFAS outcomes and binge eating scores [27,49]. This review identified that the YFAS diagnosis and symptom score explained unique variance in BED outcomes above and beyond existing measures [26,46,49]. There is significant overlap between the proposed diagnostic criteria for FA and BED as specified in the DSM-5, and there have been suggestions that FA may be a more severe variant of disordered eating [66,67]. Although a higher proportion of participants with BED met the diagnostic criteria for FA, not all participants with BED received an FA diagnosis, suggesting that FA may be distinguished from BED. Additionally, not all individuals with FA met the diagnostic criteria for an eating disorder in a recent study [36]. Further characterization of the FA construct is necessary in order to substantiate that FA is a clinical phenomenon distinct from other forms of disordered eating.

Two recently published studies have investigated relationships between the YFAS and bulimia nervosa. In one of these studies, individuals with bulimia nervosa were found to have a higher prevalence of FA diagnosis compared to individuals with BED [36]. In a second study, all participants with current diagnosis of bulimia met the YFAS diagnostic criteria for FA with an additional 30% of individuals with a history of bulimia meeting the criteria [40]. The lower prevalence observed in individuals with a history of eating disorders compared to those with a current diagnosis could possibly yield some insight into how FA could be treated, by modelling therapies on to those routinely used to treat eating disorders, such as cognitive behavior therapy. It must be noted that both BED and bulimia nervosa are associated with a pattern of excessive food consumption, sometimes coupled with compensatory behaviors, and it would be reasonable to predict that the characteristics of the proposed FA construct overlap with these conditions to some extent. However, these results require replication in other types of eating disorders such as anorexia nervosa where food restriction is the focus of disordered eating.

Only three studies assessed FA in combination with specific foods or nutrients [44,51,52]. It is unlikely that all foods are equally capable of triggering an addictive like response, yet limited research has been undertaken to examine specific foods that have been consumed in an addictive way. Individuals identified as food addicted were found to have a significantly higher intake of macronutrients including fat and protein in one study using a food frequency questionnaire to assess usual intake [51]. However, specific foods associated with FA were not reported in this study. In other included studies, cola [52], starchy foods and takeaway [44] were identified as specific foods associated with addictive food tendencies. In these studies, however, diet outcomes were assessed via the Food Cravings Questionnaire and self-reported means, whose limitations in identifying FA have been previously discussed [4]. Identification of specific foods associated with FA is important given the general population consume whole foods rather than single nutrients and data at this level could be used to inform possible treatment targets for FA, if indeed FA is found to be a clinical disorder. These results require confirmation and future studies should include using appropriate validated dietary assessment tools to identify and profile foods most associated with FA.

Only one study used a quantitative measure to assess FA using fMRI to assess whether FA scores correspond with brain activity [11]. Individuals with high FA scores were found to have comparable neural responses when viewing food images as individuals with drug dependence viewing drug cues. However, this study was limited to females exclusively and did not use the YFAS diagnostic criteria cut points. A second study used a quantitative proxy of eating behavior, the weighed amounts of snack foods consumed, to assess possible relationships with YFAS outcomes [34]. This study identified that the amount of food consumed was not reduced in food addicted individuals following the administration of an appetite suppressant. Although the YFAS has been shown to have adequate psychometric properties and associations with other eating related variables such as the Binge Eating Scale and Eating Disorder Examination [27,32,36,37,40,45,46,49], further validation of the YFAS using quantitative measures is required.

The majority of studies reported YFAS outcomes using both the diagnosis and symptom score. The mean number of symptoms reported across studies was three out of seven, which is the diagnostic cut-off for FA in combination with clinical impairment or distress. This indicates that FA characteristics derived from the application of the DSM-IV criteria to food behaviors are quite highly endorsed across populations studied to date. However, when analyzed by clinical status, it was found that the mean symptom score of studies conducted in clinical settings was more than double that of non-clinical samples, which would likely have inflated the total mean symptom score. The significance of the differences between a high symptom score without clinical impairment or distress (*i.e.*, ≥6 symptoms) compared to a lower symptom score but satisfying the criteria for diagnosis (*i.e.*, ≥3 symptoms plus clinical impairment or distress) is yet to be investigated in detail. That is, although the diagnostic criteria have been modelled from the criteria to diagnose substance dependence, potentially the symptom score could provide comparable or more valuable information regarding FA, particularly in terms of developing future treatment approaches. The most meaningful method of scoring the YFAS should be more comprehensively investigated to further standardize the reporting FA characteristics. Two studies classified as high and low FA based on YFAS scores [11,41,42] and a third study reported FA status using a numerical point score [52]. Importantly, there was no standardized approach to these alternative scoring methods, making the comparison of these studies with other studies using the predefined scoring criteria difficult.

Since the development of the original YFAS in 2009, modifications have been made to this tool for use in different populations. Five of the studies administered the YFAS via an online survey demonstrating the acceptability of the questionnaire completed online, which aids in reducing researcher and participant burden and highlights the movement towards use of technology in health assessment. Reducing the number of overall questions and subsequently reducing participant burden in the development of the m-YFAS has allowed for assessment of FA in large scale epidemiological surveys [35,50] and could potentially be used in future nationally representative samples. The assessment of addictive food behaviors at younger ages via the YFAS modified for children (YFAS-C) is important as it is well documented that child eating patterns and weight status track into adulthood [68,69]. The identification and possible treatment of FA symptoms at a young age could avoid carry-over of FA tendencies from childhood to adulthood, much like the increased risk of adult obesity associated with childhood obesity.

The results of this review should be interpreted with caution due to the inherent limitations of the YFAS tool, including use of self-reported measures and lack of accepted definition for FA. However, the YFAS does not specifically refer to the term “food addiction” thus minimizing potential bias arising from self-report. The reviewed articles were predominantly cross-sectional precluding inferences about cause and effect. A limited number and spectrum of disordered eating studies were included in meta-analysis and findings should be interpreted accordingly. This review was further constrained by the limited number of studies reporting YFAS outcomes for older adults and children exclusively, which prevented meta-analysis in these age groups. Additionally, study populations were predominantly female and obese, limiting the generalizability of findings. Prevalence of FA identified through meta-analysis is likely higher than that seen in the general population as the majority of studies were conducted in clinical settings of overweight/obese individuals. A nationally representative sample is required to provide a better estimate of addictive like-eating in the general population.

## 5. Conclusions

This study systematically reviewed all studies that used the YFAS to assess FA. Meta-analysis indicated that overweight/obese females aged over 35 years may be more predisposed to FA, as assessed by the YFAS. Additionally, participants with disordered eating had a much higher prevalence of FA, as assessed by the YFAS compared to their non-clinical counterparts. Notably, populations included in the reviewed studies were predominantly female, overweight/obese and adults aged over 35 years, and may not be representative of the general population. Further research is required to explore YFAS outcomes across a broader spectrum of ages, particularly children and adults aged >65 years, other types of eating disorders and in conjunction with weight loss interventions to confirm the efficacy of the tool to assess for the presence of FA. Additionally, future studies should investigate whether YFAS scores can be validated using a quantitative measure. This will provide further evidence to confirm or refute existence of FA and potentially help to develop appropriate treatment approaches to target FA specifically.
